# Elevated hippocampal and cortical cefepime concentrations correlate with seizures in an acute kidney injury rat model

**DOI:** 10.1128/aac.01005-25

**Published:** 2026-02-17

**Authors:** Gwendolyn M. Pais, Emily Lesnicki, Sylwia Marianski, Kimberly Valdez, Zoe Gibson, Jeffri Christopher, Kathy Lepard, Annette Gilchrist, Tomoyuki Mizuno, Marc H. Scheetz

**Affiliations:** 1 Department of Pharmacy Practice, College of Pharmacy, Midwestern University 69281, Downers Grove, Illinois, USA; 2 College of Pharmacy, Pharmacometrics Center of Excellence, Midwestern University 69281, Downers Grove, Illinois, USA; 3 College of Graduate Studies, Biomedical Science Program, Midwestern University 69281, Downers Grove, Illinois, USA; 4 College of Pharmacy, Doctor of Pharmacy Program, Midwestern University 15475, Downers Grove, Illinois, USA; 5 Department of Biomedical Sciences, College of Graduate Studies, Midwestern University 69281, Downers Grove, Illinois, USA; 6 Department of Pharmaceutical Sciences, College of Pharmacy, Midwestern University 69281, Downers Grove, Illinois, USA; 7 Department of Pediatrics, University of Cincinnati College of Medicine 12303, Cincinnati, Ohio, USA; 8 Division of Translational and Clinical Pharmacology, Cincinnati Children’s Hospital Medical Center, Cincinnati, Ohio, USA; 9 Department of Pharmacology, College of Graduate Studies, Midwestern University 69281, Downers Grove, Illinois, USA; Providence Portland Medical Center, Portland, Oregon, USA

**Keywords:** pharmacodynamics, pharmacokinetics, pharmacology, rat, toxicodynamics, cefepime, neurotoxicity, cortex, hippocampus

## Abstract

Cefepime is associated with neurotoxicity, particularly in the setting of renal impairment where drug exposure increases. This study evaluated cefepime concentrations in the brain tissue during neurotoxicity in a rat model of acute kidney injury (AKI). Male Sprague-Dawley rats (*n* = 18) received daily intravenous cefepime at 1,250 or 1,593 mg/kg for 5 days. Acute kidney injury was induced using folic acid (250 mg/kg on day 1, then 100 mg/kg/day prior to cefepime). Seizure activity was assessed using a modified Racine scale. Plasma samples were collected three to four times per day; the brain tissues (cerebral cortex and hippocampus) were collected at euthanasia. Cefepime concentrations were measured via liquid chromatography-tandem mass spectrometry (LC-MS/MS). Pharmacokinetic/pharmacodynamic (PK/PD) modeling was performed using Monolix 2024R1. Rats with seizure scores >1 had significantly higher median cefepime levels in the cerebral cortex (64.2 vs 14.1 μg/g, *P* = 0.0014) and hippocampus (66.2 vs 15.0 μg/g, *P* < 0.0001). Median (IQR) estimated exposures in the cortex were AUC_0–24_ = 565 (161.5–1,346) mg·h/L and C_max_ = 36.0 (16.3–75.6) mg/L, and in the hippocampus were AUC_0–24_ = 694.8 (151.5–1,152) mg·h/L and C_max_ = 41.9 (13.7–62.8) mg/L. Neurotoxicity in the rat correlated with plasma AUC_0–24_ > 30,000 mg·h/L and brain tissue concentration of approximately 40 mg/L. Neurotoxicity was achieved via intravenous dosing of cefepime in this rat model of acute kidney injury. Cefepime concentrations were higher in the cerebral cortex and hippocampal brain tissues in animals that had seizure stages >1.

## INTRODUCTION

Cefepime is a broad-spectrum cephalosporin used to treat bacterial infections, such as pneumonia, urinary tract infections, and skin infections, commonly caused by gram-positive and -negative bacteria (1). It is the fourth most commonly used gram-negative antibiotic administered to hospitalized patients. While a class effect of neurotoxicity is known for β-lactam agents (2), cefepime, in particular, is associated with a high rate of neurotoxicity. Comparative preclinical studies indicate that cefepime exhibits approximately 1.6-fold higher pro-convulsant activity than penicillin and nearly double that of imipenem, whereas ceftriaxone is 10 times less pro-convulsant (3). Yet, the precise cefepime plasma concentration thresholds leading to neurotoxicity remain unclear. Some have suggested trough concentrations > 22 mg/L are a driver (4); however, this concentration is regularly reached, and we previously estimated that up to 51.4% of patients, on some dosing schemes, would reach this threshold (making it unlikely that this is truly the threshold for toxicity) (5). Meanwhile, it does appear clear that patients with reduced kidney function are at a greater risk for neurotoxicity. In 2012, the Food and Drug Administration (FDA) issued a warning of seizure risk associated with cefepime use in patients suffering from renal impairment who do not receive appropriate dose adjustments (6). Of the 59 individuals displaying neurotoxic outcomes while on cefepime therapy, 58 of those patients had renal dysfunction, and 56 patients received a higher than recommended cefepime doses for their organ function.

Roughly 86% of the cefepime is recovered in the urine unchanged in patients with normal renal function (7). When the blood–brain barrier is disrupted, greater concentrations of the drug are likely to reach the brain. Increased central nervous system (CNS) penetration has also been observed in patients with sepsis, CNS infection, and brain injury (8). While there are data on penetration of cefepime in CSF and plasma, little is known about accumulation in the brain and relationship with toxicity. Thus, the objectives of the current research are to define cefepime pharmacokinetic exposures that are associated with neurotoxicity, specifically in the context of renal impairment, using a validated rat model.

## MATERIALS AND METHODS

This PK-TD study was conducted at Midwestern University, Downers Grove, Illinois, with the approval of the Institutional Animal Care and Use Committee (IACUC protocol #2793). All experiments were conducted in compliance with the National Institutes of Health *Guide for the Care and Use of Laboratory Animals* (9).

### Chemicals and reagents

Animals were administered clinical-grade cefepime hydrochloride for injection (Sagent Pharmaceuticals, Schaumburg, IL, USA). Normal saline (Veterinary 0.9% Sodium Chloride Injection USP, Abbott Laboratories, North Chicago, IL, USA) and heparin (Covetrus, Portland, ME, USA) were used during surgery and sampling procedures. Folic acid (Sigma-Aldrich, St. Louis, MO, USA) dissolved in sodium bicarbonate (VWR, Radnor, PA, USA) was administered intraperitoneally to induce renal dysfunction.

For assay work, analytical-grade cefepime hydrochloride (Sigma-Aldrich Co, St. Louis, MO, USA, Lot #LRAA9570) and ceftazidime pentahydrate (Acros Organics, NJ, USA, Lot #A0390536) as an internal standard were used for liquid chromatography-tandem mass spectrometry (LC-MS/MS) assays. Milli-Q water was obtained from Aqua Solutions purified water dispensing system at Midwestern University. LC-MS/MS-grade acetonitrile, formic acid, and methanol were obtained from VWR International (Radnor, PA). Frozen, non-medicated, non-immunized, pooled Sprague-Dawley rat EDTA plasma was purchased from BioIVT, Westbury, NY, and frozen brain cortex and hippocampus from untreated control rats were used as a blank matrix for the generation of LC-MS standard curves.

### Experimental design and animals

**Fig 1:**
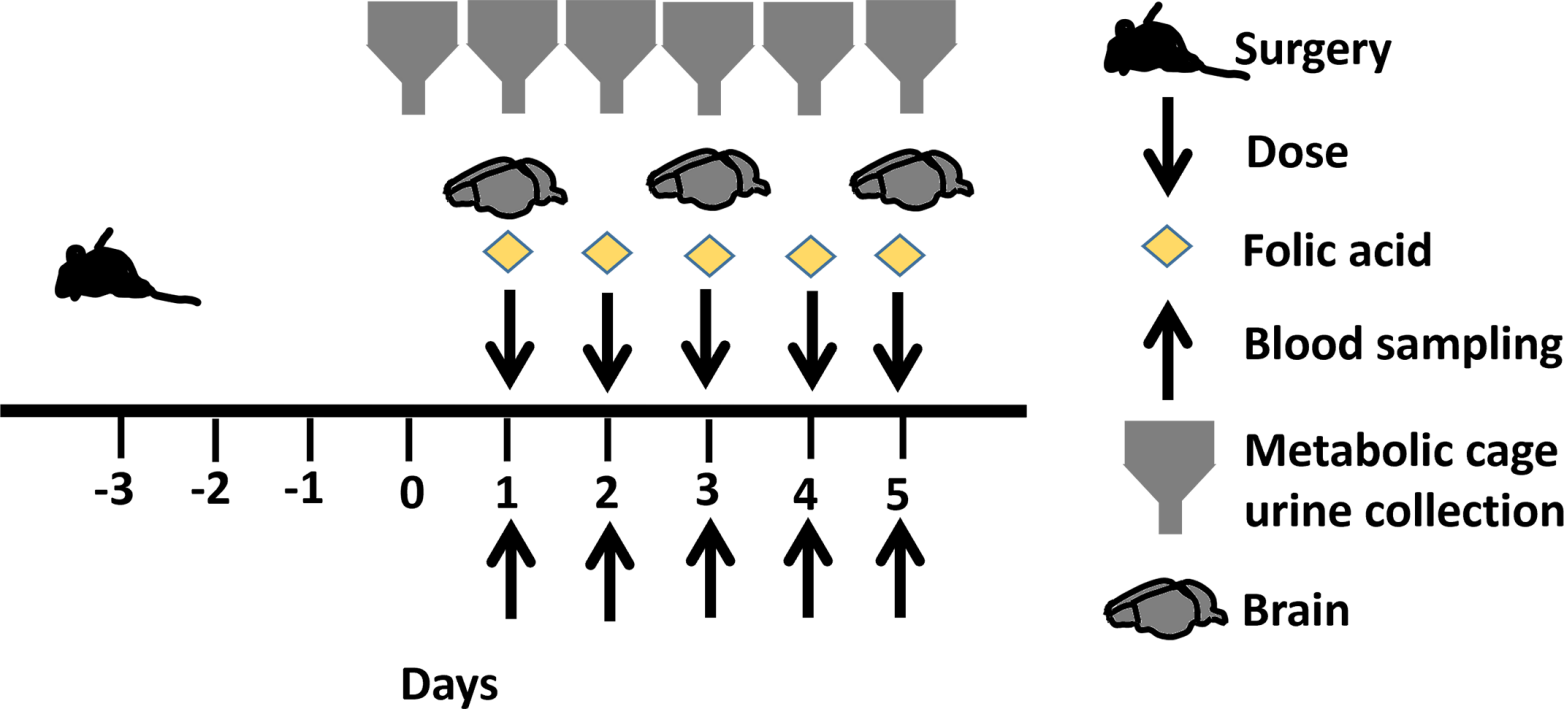
Experimental timeline.

Male Sprague-Dawley rats (*n* = 18, approximately 9 to 10 weeks old; mean [±SD] weight, 282 [±17.0] g, Envigo, Indianapolis, IN, USA) were housed in open cages in pairs (prior to surgery) and singly (post-surgery) in a light- and temperature-controlled room. They were allowed free access to water and food for the duration of the study. Animals underwent double jugular catheterization 72 h prior to protocol initiation. Following recovery from surgery, animals were transferred to metabolic cages (Nalgene, Rochester, NY) for 24-h urine collection, 24 h pre-study (day 0), and on days 1–4 (Fig. 1).

Dosing for experiments was determined based on two preliminary studies (10) conducted by us (and briefly described below). In the first study, *n* = 3 rats received 531 mg/kg/day of cefepime i.v. over approximately 2 min. This was an allometrically scaled dose based on the maximum human total daily dose of 86 mg/kg/day for a 70 kg patient. The FDA-approved dose of 2 g three times daily was converted to a weight-based dose using a standardized patient weight of 70 kg to obtain the maximum human total daily dose (1). Animals served as their own controls. Plasma samples were collected at various times before and after a single folic acid dose of 250 mg/kg (11) to mimic pre- and post-acute kidney injury (AKI) conditions. Folic acid increased the plasma elimination half-life of cefepime by a factor of 10-fold, which is similar to humans with renal dysfunction (1). In all studies, folic acid was administered intraperitoneally (i.p.) under isoflurane (5% induction, 2–3% maintenance), and cefepime was administered 30 min post-folic acid injection.

Using a single folic acid dose of 250 mg/kg to induce AKI, we then carried out a cefepime dose escalation and maximum tolerated dose (MTD) study according to FDA guidance (12). Cefepime dose was escalated from 531 mg/kg/day to 2,124 mg/kg (4× the total daily dose in humans) administered as i.v. infusions at a rate of 0.5–1 mL/min (*n* = 1). Folic acid was not administered on subsequent days. The second group (*n* = 1) received the maximum dose, and loss of life at an average time of 4.3 min post-dose was observed. With 1,593 mg/kg (3× the total daily dose in humans based on AUC), no loss of life was observed; seizure stages 1–6 were recorded, and time to seizure varied from 1 to 4 h; hence, 1,593 mg/kg was determined to be the MTD.

From these preliminary experiments, we set the folic acid dose to 250 mg/kg on day 1 and 100 mg/kg daily (13) thereafter to induce AKI and recreate the clinical scenario in which cefepime neurotoxicity is regularly observed. In the present study, rats received either a single dose of folic acid 250 mg/kg and were sacrificed on day 1 either at the onset of seizure or at predetermined time points or multiple doses of folic acid (250 mg/kg on day 1, followed by 100 mg/kg daily thereafter). The maximum tolerated dose of cefepime (either 1,593 mg/kg once or 1,250 mg/kg daily) was administered intravenously (i.v.) via a dedicated jugular vein catheter 30 min post-folic acid injection. Convulsive behavior was assessed by a modified Racine scale (14): stage 0, no response; stage 1, ear and facial twitching; stage 2, myoclonic body jerks; stage 3, forelimb clonus and rearing; stage 4, clonic convulsions and turned on the side; stage 5, generalized clonic convulsions and turned on the back; stage 6, status epilepticus; and stage 7, loss of life. The experimental timeline is outlined in Fig. 1. Serial sacrifice occurred at 30 and 120 min on day 1 and at 120 min on days 3 and 5 either before or during convulsive episodes. Seizure stages were determined by the last observed seizure before brain harvest, and brain tissue concentrations are expressed as µg/g of the brain tissue.

### Sample collection

Blood samples (0.15 mL at 0, 15, 30, and 120 min) were drawn from a dedicated jugular vein catheter into EDTA-containing tubes and centrifuged at 3,000 × *g* for 10 min at 4°C, and the resultant plasma was stored at −80°C for batch analysis. Samples were replaced with an equivalent volume of normal saline to maintain euvolemia. Dilute heparin (0.1 mL) was administered to prevent clotting. Urine was collected continuously, aliquoted every 24 h, centrifuged (500 × *g*, 10 min at 4°C), and the supernatant was stored at −80°C. At the end of the protocol, animals were anesthetized with ketamine/xylazine (100/10 mg/kg, by intraperitoneal injection). Tissues were perfused with chilled saline to prevent contamination with residual blood, and brains were harvested, rinsed in cold saline, flash frozen, and stored at −80°C for cefepime assay.

### Tissue homogenization

A BeadBug (Benchmark Scientific, Sayreville, NJ) tissue homogenizer was used to homogenize cerebral cortex and hippocampus samples. Brains were thawed on ice and micro-dissected. Approximately 100 mg of cortex and hippocampus was placed into screw cap tubes, matched to an exact 1:3 ratio of sample weight to volume of MilliQ, and filled with 3.0 mm zirconium beads. Tissues underwent 3 cycles at 2,500 rpm for 30 s with a 30-s rest interval repeated twice. The homogenate was centrifuged twice (16,000 × *g*, 10 min, 4°C), and the resultant supernatant was stored at −80°C for batch analysis.

### Estimation of cefepime in plasma and brain homogenates

Cefepime concentrations in the plasma, cortex, and hippocampus were analyzed by LC-MS/MS similar to our previous report (15), with minor noted exceptions. Due to high cefepime concentrations in plasma, samples were diluted (i.e., 136, 62, 32, 8, or 4×) with corresponding matrix, so concentrations were within the standard curve range. Briefly, standard curves were prepared using fresh cefepime and ceftazidime. Plasma or tissue homogenate volumes of 40 µL were combined with 4 µL of internal standard (100 µg/mL ceftazidime) and subject to protein precipitation using 456 µL of 0.1% formic acid in methanol. Samples were centrifuged at 16,000 × *g* for 10 min at 4°C, and 100 µL supernatant was collected for analysis. The plasma and tissue concentrations were quantified by LC-MS/MS using standard curves for each matrix. Milli-Q water containing 0.1% formic acid and acetonitrile (flow rate of 0.45 mL/min) was used as aqueous (A) and organic (B) solvents, respectively, at the following ramping transitions: 1.50 min A (90%) → B (10%), 2.50 min A (10%) → B (90%), 5.49 min A (10%) → B (90%), 5.50 min A (90%) → B (10%), and 10 min A (90%) → B (10%). A Waters (2.1 × 100 mm, 1.7 μm) Acquity UPLC CSH C18 column (Waters Corporation, Taunton, MA, USA) was utilized. The following transitions (*m*/*z*) for cefepime and ceftazidime were utilized: 241.1 → 84.1 for cefepime quantifier, 241.1 → 86.1 for cefepime qualifier, and 274.1 → 80.2 for ceftazidime quantifier. The assay was linear between plasma concentrations of 0.5 and 100 μg/mL (*R*^2^ = 0.996) and brain tissue concentrations of 0.5 and 80 μg/mL (*R*^2^ = 0.998). Imprecision was <5% for all inter- and intra-assay measurements. Mean assay accuracies were 100% for the plasma and 99.2% for the brain tissue concentrations.

### Cefepime PK model and estimation of PK exposures in the brain tissues

Plasma and tissue concentrations were used to develop a PK model in Monolix 2024R1 (Lixoft) using the stochastic approximation expectation-maximization (SAEM) algorithm (16) for parameter estimation. Concentrations below the limit of quantitation were interval censored in Monolix. Specifically, the likelihood of these measurements was estimated and assumed to be positive. Multiple PK models of cefepime transit between plasma and brain compartments (i.e., cerebral cortex and hippocampus) were explored. The best-fit model was utilized to obtain median PK parameters. Exposures during the first 24 h (i.e., area under the concentration-time curve from 0 to 24 h [AUC_0–24_] and maximum concentration of drug in plasma from 0 to 24 h [Cmax_0–24_]) were calculated from empirical Bayes estimated concentrations given exact dosing amounts and infusion schedules for each rat in Simulx (Lixoft). The Cmax in the central, cortex, and hippocampus compartments was estimated from the Bayesian posterior profiles for each animal in 6-min intervals. Model performance was evaluated using observed versus predicted concentrations. The final model was selected according to the lowest Akaike information criterion (AIC) score and the rule of parsimony. PK parameters from the brain tissue were correlated with convulsive behavioral scores (i.e., modified Racine scale scores) (14).

### Statistical analysis

All statistical analyses and graph generation were conducted using GraphPad Prism 10 (GraphPad Software, Inc., La Jolla, CA) or Stata BE 17.0 (StataCorp LLC, College Station, TX). Median cefepime concentrations and PK exposures in the plasma, cortex, and hippocampus between seizure stage groups (≤1 vs. >1) were compared using the Mann-Whitney test. Logistic regression models were used to assess the relationship between seizure stage >1, the probability of reaching seizure stage >1, and cefepime exposure. The exposure associated with a 50% probability of seizure was used as the cut-off for Kaplan-Meier (KM) analyses. Survival analysis was performed using the KM method to estimate and visualize the cumulative probability of AUC exposure over time. Animals were grouped according to the AUC_plasma cut-off value (below vs. above 30,658.6 mg.h/L). Time to seizure was measured from start of cefepime treatment. All tests were two-tailed, with the statistical significance set at alpha 0.05.

## RESULTS

### Cefepime accumulation in the brain

**Fig 2:**
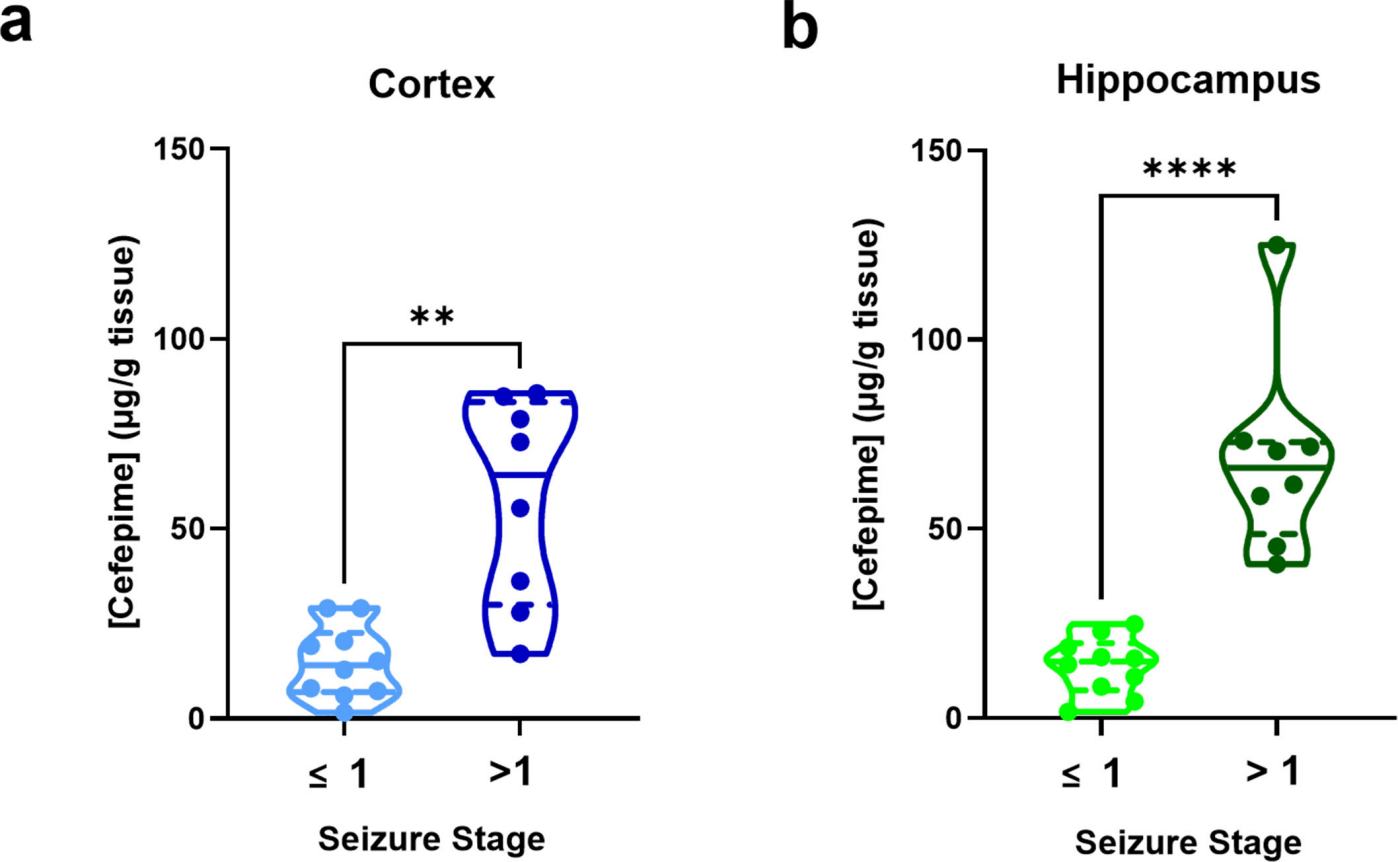
Measured concentrations of cefepime in the rat cerebral cortex and hippocampus at euthanasia relative to seizure stage. Cefepime concentrations expressed as µg/g brain tissue. Concentrations in the (**a**) cerebral cortex and (**b**) hippocampus were higher in rats exhibiting seizure stages >1 (**P = 0.0014 and ***P < 0.0001, respectively, by Mann-Whitney test) compared to rats exhibiting seizure stages ≤1.

Rats exhibiting seizure activity (stages >1) demonstrated a significantly higher cefepime concentration in both the cerebral cortex and the hippocampus compared to rats with no convulsive symptoms. Median (IQR) cefepime concentrations in the cortex for the seizure group were 64.2 (30.0–83.4) vs 14.1 (7.0–22.6) μg/g in the non-seizure group (*P* = 0.0014). Similarly, hippocampal concentrations were 66.2 (47.7–72.9) vs. 15.0 (7.4–19.8) μg/g, *P* < 0.0001, Fig. 2.

### Cefepime pharmacokinetic model and estimated exposures

A total of 18 rats received cefepime for a total of 129 plasma, 18 cortex, and 18 hippocampal tissue samples. Of these, 127 plasma and all available brain tissue samples were used in model building and analysis. Two rats had a single plasma concentration that was 3 standard deviations (SD) from the mean; it was unclear why this occurred, but these concentrations were excluded from the analysis.

**Fig 3:**
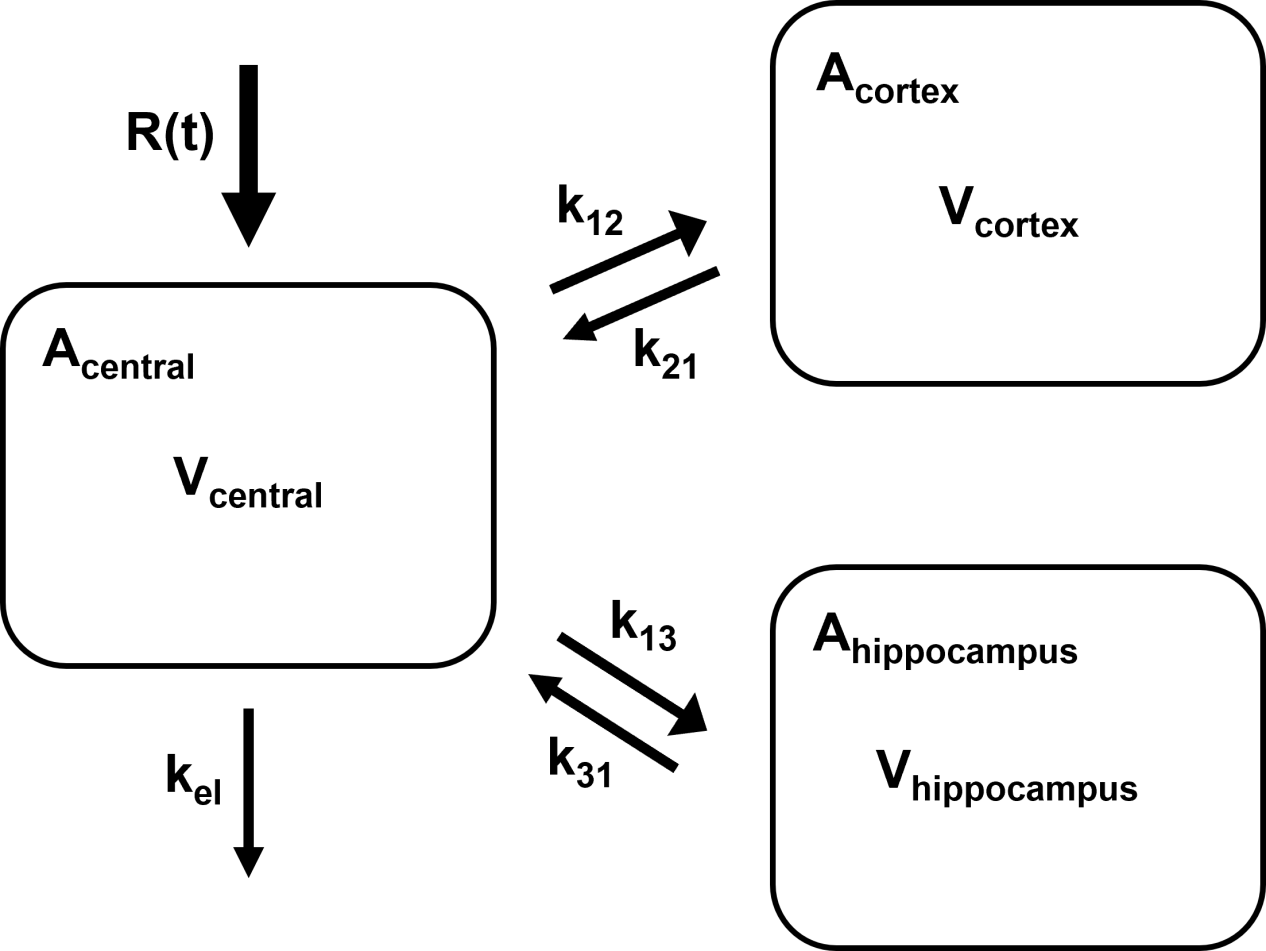
Schematic and differential equations of the three-compartmental PK model. Abbreviations: PK, pharmacokinetic; R(t), dose administration rate; k_el_, elimination rate constant; V_central_, volume of the central compartment; V_cortex_, volume of the cerebral cortex compartment; V_hippocampus_, volume of the hippocampus compartment; k_12_, rate constant to the cortex from the central compartment; k_21_, rate constant to the central compartment from the cortex; k_13_, rate constant to the hippocampus compartment from the central compartment; k_31_, rate constant to the central compartment from the hippocampus compartment; A_central_, amount in the central compartment; A_cortex_, amount in the cortex compartment; and A_hippocampus_, amount in the hippocampus compartment.

We created models with the fewest number of sampled anatomic locations, models with additional volume of distribution, and models that incorporated various transfer considerations, such as a lag compartment (Table 1). The final model was a three-compartmental PK model for plasma, cerebral cortex, and hippocampus allometrically scaled to the median rat weight of the data set (Fig. 3). The median parameter values with the relative standard error percentage (RSE%) for the rate constants to the cerebral cortex from the central compartment (k_12_), to the central compartment from the cerebral cortex compartment (k_21_), to the hippocampus from the central compartment (k_13_), to the central compartment from the hippocampus compartment (k_31_), the elimination rate constant (kel), and the volumes of distribution in the central compartment, cortex, and hippocampus are listed in Table 2. In the final model, a significant correlation was found between the random effects of the elimination rate constant and the volume of distribution in the hippocampus (*R*^2^ = 0.92). Since this correlation improved the model (Table 1), it was retained.

**TABLE 1:** Pharmacokinetic model build comparison^*a*^

Model	−2LL	BIC
Three-compartment^*b*^	2,249.5	2,322.2
Three-compartment (fixed k_12_)	2,248.0	2,315.5
Three-compartment (corr V_hippocampus_k_el_)	2,242.2	2,317.8
Three-compartment (fixed k_12_ and corr V_hippocampus_k_el_)	2,241.4	2,311.9
Four-compartment with peripheral compartment (k_14_ and k_41_)	2,246.7	2,332.5
Four-compartment with lag compartment to hippocampus (k_14_ and k_43_)	2,247.6	2,325.3

^*a*^ Abbreviations: k_el_, elimination rate constant from the central compartment; k_14_, rate constant to the lag compartment or to the peripheral compartment from the central compartment; k_41_, rate constant to the central compartment from the peripheral compartment; k_43_, rate constant to the hippocampus from the lag compartment; −2LL, −2 log likelihood; and BIC, Bayesian information criterion.

^*b*^ Final model based on regression of observed versus predicted concentrations, visual plots of parameter estimates, lowest BIC, and rule of parsimony.

### Ordinary differential equations


$$ddt\_A_{central}=-(k_{el}+k_{12}+k_{13})*A_{central}+(A_{cortex}*k_{21})+(A_{hippocampus}*k_{31})$$

$$ddt\_A_{cortex}=k_{12}*A_{central}-(A_{cortex}*k_{12})$$

$$ddt\_A_{hippocampus}=k_{13}*A_{central}-(A_{hippocampus}*k_{31})$$

$$C_{central}=A_{central}/V_{central}$$

$$C_{cortex}=A_{central}/V_{cortex}$$

$$C_{hippocampus}=A_{hippocampus}/V_{hippocampus}$$

**TABLE 2:** Median parameter values from the final model^*a*^

PK parameter	Median	RSE%
k_el_ (h^−1^)	0.14	38.7
beta_kel_logtWT	0.75	
V_central_ (L)	0.12	6.85
beta_V_central__logtWT	1	
V_cortex_ (L)	0.0039	18.6
V_hippocampus_ (L)	0.00039	14.8
k_12_ (h^−1^)	0.00013	
k_21_ (h^−1^)	0.17	18.5
k_13_ (h^−1^)	0.000015	24.9
k_31_ (h^−1^)	0.15	31.2
**Correlations**		
corr_k_el__V_hippocampus_	0.92	20.8

^*a*^ Abbreviations: PK, pharmacokinetic; RSE%, relative standard error percentage; k_el_, elimination rate constant; beta_k_el__logtWT, beta coefficient of the log-transformed weight and elimination rate constant; V_central_, volume of distribution in the central compartment; V_cortex_, volume of distribution in the cortex; V_hippocampus_, volume of distribution in the hippocampus compartment; beta_V_central__logtWT, beta coefficient of the log-transformed weight and volume of distribution in the central compartment; k_12_, rate constant to cortex from the central compartment; k_21_, rate constant to the central compartment from the cortex; k_13_, rate constant to the hippocampal compartment from the central compartment; and k_31_, rate constant to the central compartment from the hippocampus.

**Fig 4:**
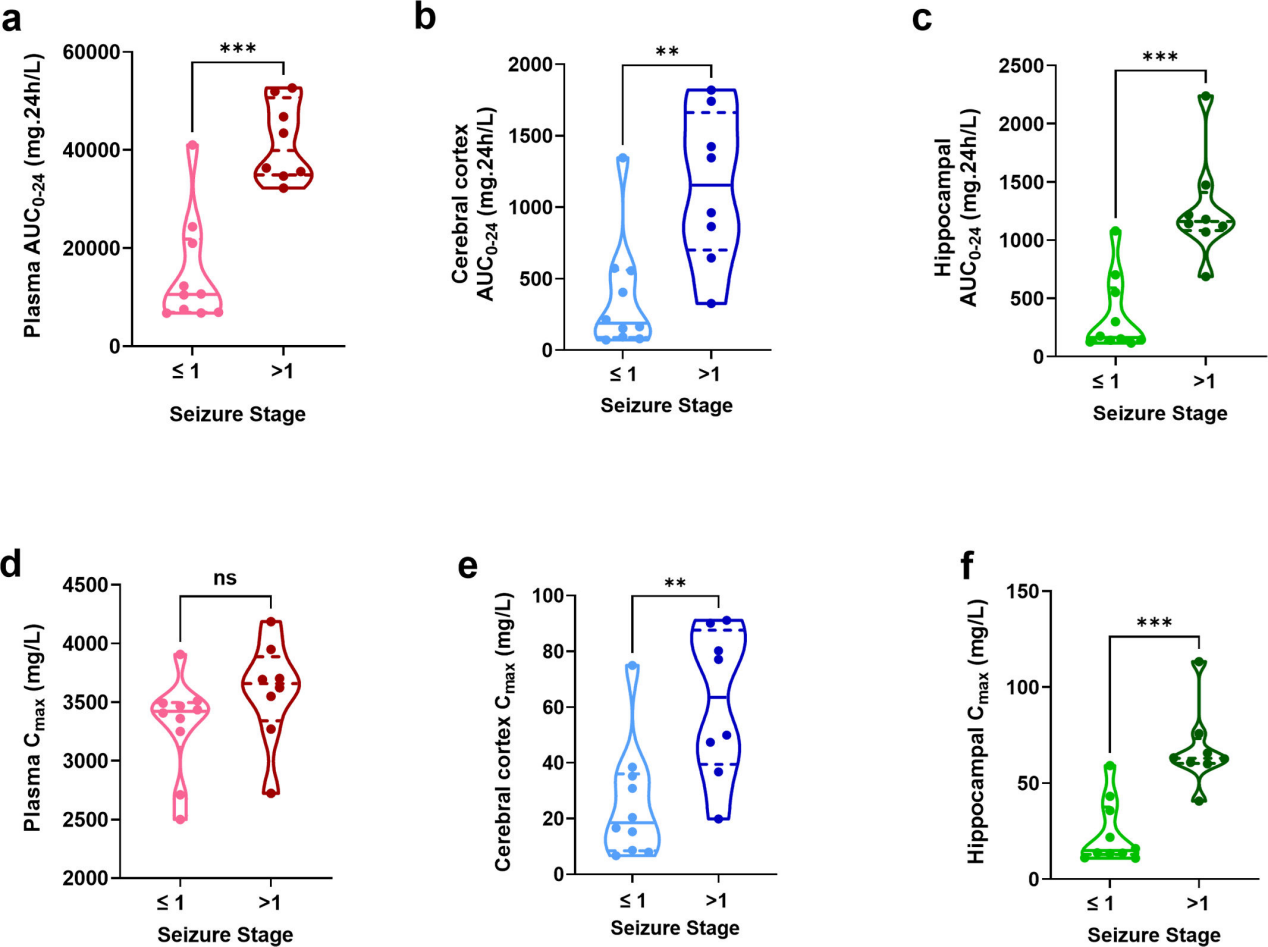
PK parameters and exposures during the first 24 h calculated from empirical Bayes estimated concentrations estimated every 0.1 h given exact dosing schedules for each rat. Cefepime AUC_0–24_ (mg·h/L) in (**a**) plasma, (**b**) cerebral cortex, and (**c**) hippocampus was significantly higher in rats exhibiting seizure stages >1 (***P = 0.0005, **P = 0.0021, and ***P = 0.0003, respectively, by Mann-Whitney test) compared to rats exhibiting seizure stages ≤1. Cefepime Cmax_0–24_ concentrations in the (**d**) plasma were not significantly different, however, cefepime Cmax_0–24_ concentrations in the (**e**) cerebral cortex and (**f**) hippocampus were significantly higher in rats exhibiting seizure stages >1 (**P = 0.004 and ***P = 0.0002, respectively, by Mann-Whitney test) compared to rats exhibiting seizure stages ≤1 ns, not significant.

Exposure estimation revealed a plasma median (IQR) AUC_0–24_ and C_max 0–24_ of 28,314 (9,737–41,639) mg· h/L and 3,480 (3,266–3,695) mg/L from the first dose, respectively. Exposure estimation of the cerebral cortex demonstrated a median (IQR) AUC_0–24_ and C_max 0–24_ of 565 (161.5–1,346) mg·h/L and 36.0 (16.3–75.6) mg/L, respectively. Exposure estimation of the hippocampus demonstrated a median (IQR) AUC_0–24_ and C_max 0–24_ of 694.8 (151.5–1,152) mg·h/L and 41.9 (31.7–62.8) mg/L, respectively. PK exposures for the first 24 h are described in Fig. 4.

**Fig 5:**
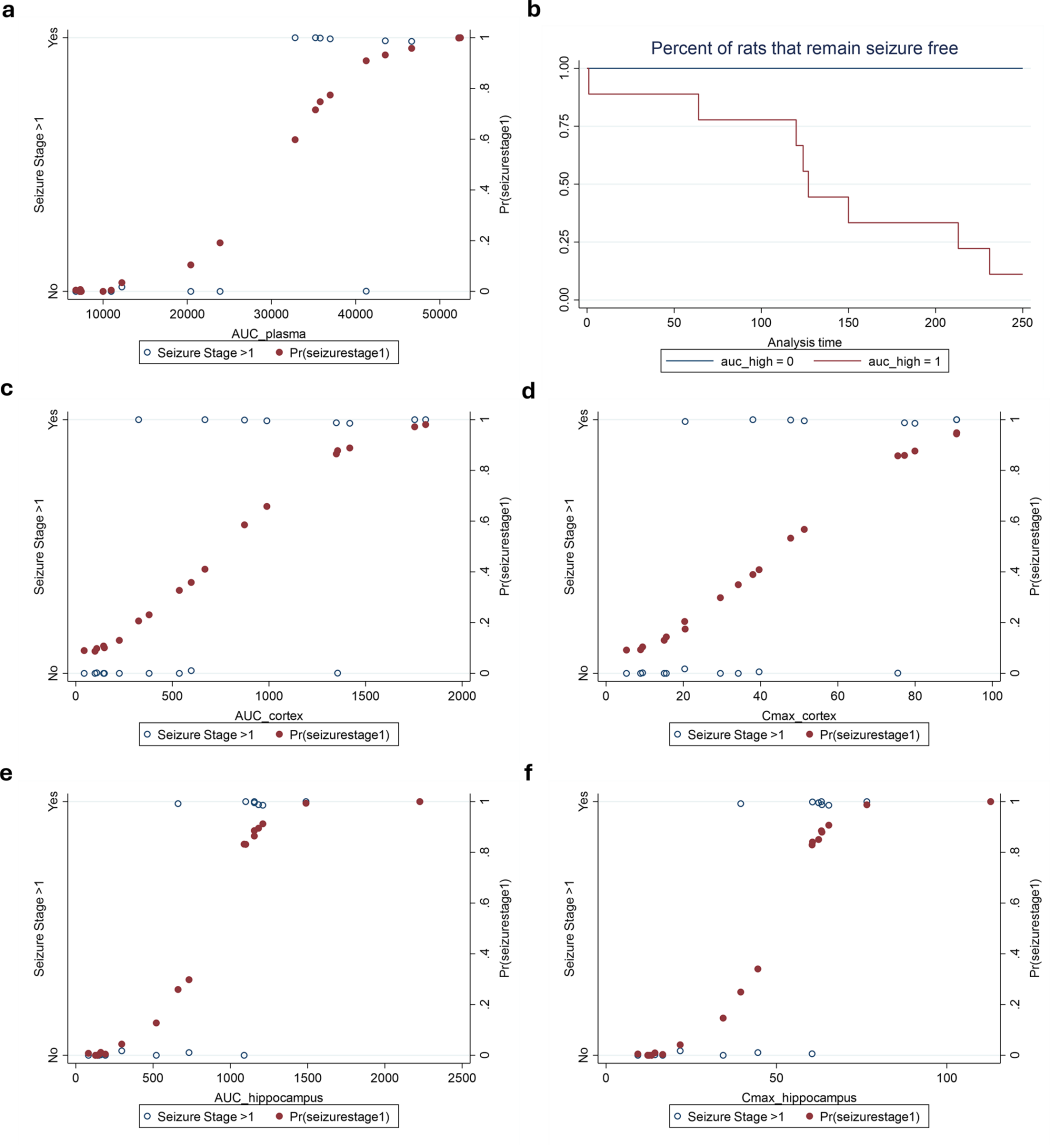
Logistic models relating seizure probability and time-to-seizure to continuous exposure metrics. Logistic models relating seizure stage >1 and probability of seizure stage >1 with plasma AUC, brain Cmax, and brain AUC (**a, c–f**). TC_50_ for exposures in the plasma, cortex, and hippocampus were 30,658.6, 760.8, and 838.2 mg.h/L, respectively, and in terms of Cmax were 45.0 mg/L in the cortex and 48.2 mg/L in the hippocampus. Kaplan–Meier curve depicting the relationship between plasma exposure and time to seizure (**b**). A cut-off of 30,658.6 mg.h/L was used for plasma AUC.

Logistic models relating seizure stage >1 and probability of seizure stage >1 with PK model derived plasma AUC, brain Cmax, and brain AUC were applied (Fig. 5a and c through f). The 50th percentile toxicity concentration (TC_50_) for exposures in the plasma, cortex, and hippocampus were 30,658.6, 760.8, and 838.2 mg.h/L, respectively, and in terms of Cmax were 45.0 mg/L in the cortex and 48.2 mg/L in the hippocampus. We plotted a Kaplan-Meier curve for the relationship between plasma exposure cut point and time to seizure, as plasma is the most commonly sampled biomatrix (Fig. 5b).

**Fig 6:**
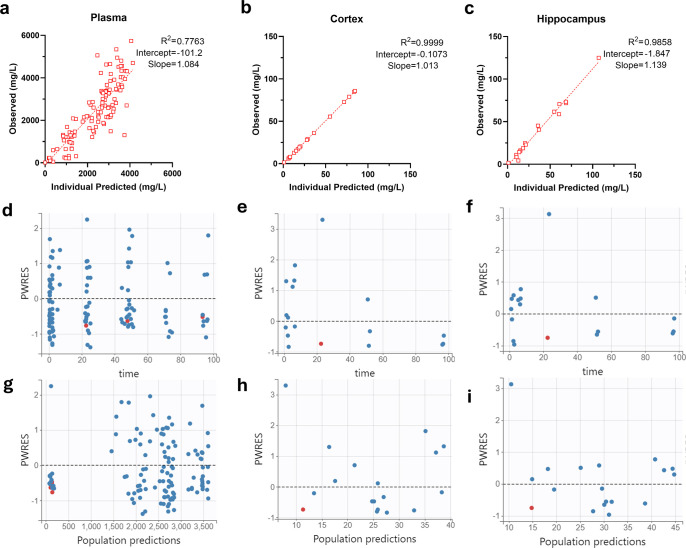
Goodness of fit plots. Observed versus predicted Bayesian plots from the final model for the (**a**) plasma, (**b**) cerebral cortex, and (**c**) hippocampus. Population-weighted residuals (PWRES) vs. time after dose for the (**d**) plasma, (**e**) cerebral cortex, and (**f**) hippocampus. PWRES vs. population-predicted concentrations for the (**g**) plasma, (**h**) cerebral cortex, and (**i**) hippocampus.

The coefficients of determination (*R*^2^) for the observed versus Bayesian predicted concentrations in the plasma, cerebral cortex, and for the hippocampus were 0.78, 0.99, and 0.985, respectively (Fig. 6a through 6c). Plots of population weighted residuals against time after the last dose and population weighted residuals against the population-predicted concentration were evenly centered around zero without systematic bias, and most values were within −2 to +2 SDs (Fig. 6d through 6i). Concentration-time profiles for the plasma, cortex, and hippocampus in the rat (Fig. S1a through c) are included to provide context for the high AUC and Cmax values in the rat.

## DISCUSSION

We induced kidney injury in rats and identified important exposure response relationships between cefepime and seizure stage. We found that the estimated AUC_0–24_ and Cmax_0–24_ exposures in the cortex and the hippocampus were significantly higher for animals experiencing neurotoxic outcomes. A cefepime plasma AUC_0–24_ around 30,000 mg·h/L and an associated Cmax of ~40 mg/L in the cortex and hippocampus were observed in animals exhibiting seizure stages >1. These plasma AUC values are 17-fold higher than the clinical (i.e., human) exposures of cefepime (17). These findings support a strong link between cefepime brain exposure and convulsive neurotoxicity in the setting of renal impairment.

Previous preclinical models have been conducted to assess cefepime neurotoxicity. In those studies, seizure activity with cefepime was induced either by lowering the seizure threshold chemically (with pentylenetetrazol [PTZ], a GABA inhibitor) or electrically (using corneal kindled mice [14] or with cefepime that was administered intracerebrally [18]); however, we believe this is the first pre-clinical report in which cefepime-induced neurotoxicity was produced using systemically (i.e., intravenously) administered cefepime. Importantly, patients without apparent seizure threshold lowering agents or conditions often develop cefepime-induced neurotoxicity. The mechanism for the CNS effects of cefepime remains unclear. The proposed explanation is attributed to its ability to cross the blood–brain barrier to bind competitively to the GABAergic receptor to suppress inhibitory neurotransmission (18). We observed substantial cefepime accumulation in the hippocampus, a brain region critically involved in seizure generation. Our proposed model recapitulates the clinical scenario of toxicity to allow the study of causative factors. Future work will be required to better understand the full relationships between plasma concentrations, various brain tissue concentrations, the effect modification of acute kidney injury, and neurotoxicity. Cefepime brain concentration findings in humans are more rare, as tissue is difficult to obtain. However, the penetration of cefepime in the CSF has been reported (4–34% based on AUC over a 12 h dosing interval) and appears similar to other beta lactams, but can vary among patients reaching as high as 97% (based on a calculated AUC over 16 h) (19).

Renal impairment is a known risk factor of cefepime neurotoxicity. In this study, we induced acute kidney injury (AKI) by administering folic acid. Folic acid induction is a model for drug-induced nephrotoxicity characterized by tubular epithelial cell injury and activation of the inflammatory system (20). This model recapitulates drug-induced acute kidney injury. A single intraperitoneal injection of high-dose folic acid in rats produces renal injury reflected by elevated BUN and SCr on day 2, followed by spontaneous recovery, with most renal function parameters returning to baseline within 5–7 days (21–23). In the present study, we used 250 mg/kg folic acid dissolved in 0.3 mM sodium bicarbonate, consistent with folic acid-induced AKI protocols in mice (20). This approach increased the plasma elimination half-life of cefepime by a factor of 8–10-fold (10), which is similar to humans with renal dysfunction, where cefepime half-life can be 6–10-fold greater (1); thus, our model yields a 10-fold prolonged cefepime half-life vs. those without kidney injury (Table S1). To prolong renal injury, we also administered a low dose of folic acid (100 mg/kg) daily (13).

Compared with other AKI models, such as glycerol-induced rhabdomyolysis, cisplatin nephrotoxicity, or adenine-induced chronic injury, the folic acid model is simple, environmentally benign, cost-effective, non-surgical, and relatively reproducible, producing robust proximal tubule injury without affecting other organs (11, 24).

Because cefepime is predominantly eliminated by the kidneys, patients with impaired renal function experience increased systemic exposure if doses are not appropriately reduced. In patients with kidney disease, the cefepime half-life increases to 13 h, substantially longer than in patients with normal clearance (7). Kidney impairment is also the most common comorbidity associated with cefepime neurotoxicity. Consistent with this, our preliminary experiments (data not shown) demonstrated that rats did not become neurotoxic in the absence of kidney injury.

Notably, we observed a significant positive correlation between the random effects (interindividual variability) of the elimination rate constant and the volume of distribution in the hippocampus. This suggests that these parameters are not independent: individuals who tend to eliminate the drug more rapidly also tend to have higher hippocampal volumes of distribution. Biologically, this may be plausible, as a larger volume of distribution in the hippocampus would likely result in lower local drug concentrations. A physiological connection may also exist, as prior research has demonstrated meaningful links between kidney function and hippocampal integrity. Both acute kidney injury (AKI) and chronic kidney disease (CKD) have been associated with hippocampal damage, reduced volume, and impaired function, contributing to cognitive decline and memory deficits (25). MRI studies further indicate that reduced kidney function (eGFR < 75 mL/min/1.73 m²) is an independent predictor of hippocampal atrophy (26).

In the present study, cefepime concentrations in both brain and plasma are reported as total concentrations. However, it is the unbound (free) fraction that is pharmacologically active and capable of crossing the BBB, interacting with GABA_A_ receptors on chloride ion channels (3), and thereby potentially lowering the seizure threshold. Cefepime protein binding has traditionally been reported as approximately 20% (27, 28), leaving roughly 80% unbound. Acute kidney injury (AKI) along with inflammation and hypoalbuminemia would result in an increase in the unbound fraction of cefepime. In the presence of a compromised BBB, higher unbound cefepime levels could more readily penetrate brain tissue and thereby elevate seizure risk. Although unbound concentrations were not directly measured in this study, these physiologic considerations support the relevance of total concentrations while highlighting the potential impact of protein and tissue binding on cefepime neurotoxicity.

Pre-existing CNS disorders are another recognized risk factor for cefepime-induced neurotoxicity (29). It is plausible that such conditions alter blood–brain barrier permeability and lower the seizure threshold (4, 14, 30–33). The present study was designed to measure plasma and brain tissue concentrations reflective of patients with AKI. CSF concentrations were not collected because placing cisternal catheters for sampling can itself lower the seizure threshold, thereby introducing an additional confounding variable.

No gender-related differences in cefepime pharmacokinetic parameters have been reported (34) and therefore, only male rats were used in this study. However, future work studying the neurotoxic effects of cefepime should account for sex as a biological covariate.

There are several limitations in this study. First, in the final model, the transfer rate constant from the central compartment to the cortex was fixed to reduce RSE%. Precise estimation is difficult unless in the setting of rich sampling. Rich sampling is difficult because obtaining brain samples requires sacrifice, unless microdialysis is performed (which was out of scope in the present study). Further explorations will be necessary to determine parameter estimates more precisely. Second, in our study, only single daily doses of cefepime were administered to healthy animals, so it remains unclear whether multiple daily doses would produce concentration-dependent changes in cerebral cortex and hippocampal transit. Additionally, because cefepime is indicated for infections, such as sepsis and meningitis, and inflammation increases the permeability of biological barriers, including the blood–brain barrier (35, 36), treatment under inflammatory conditions with a multidose regimen may alter brain exposure and thereby influence neurotoxicity risk. Third, we did not study lipid metabolism here, which is a proposed mechanism. Because the brain is a lipid-rich organ, dysregulated homeostasis may contribute to the development of cefepime neurotoxicity (37). Cefepime has been found to dysregulate the glycerophospholipid profile in the corpus striatum in mice receiving intraperitoneal injection. The number of dysregulated lipids increased after 5 days of exposure, and changes in composition and structure were also observed. Moreover, the proportion of GABAergic neurons is high in the cortex and hippocampus but may be higher within the striatum (37). This area may be more sensitive to cefepime treatment. Our study did not have adequate brain samples remaining to isolate and analyze the corpus striatum. Further studies are warranted. Fourth, seizure scoring using the modified Racine scale was not blinded to treatment, which could introduce potential bias in seizure stage assessment. In the present study, dichotomization (i.e., any seizure activity or not) helped reduce observer-related variability. Additionally, in humans, cefepime neurotoxicity most commonly manifests as non-convulsive status epilepticus rather than overt convulsive activity. Therefore, separating stages into non-convulsive (≤1) and convulsive (>1) allowed us to present the findings in a way that is clinically meaningful. At the present time, funding was not sufficient to complete a different study design. Future studies will benefit from incorporating video monitoring to allow for blinded, retrospective scoring and to more accurately capture both time to seizure onset and seizure severity. Finally, brain histology was not performed to corroborate neurotoxicity. c-Fos staining, which is widely used as a marker of neuronal activity, can be combined with neuronal and glial markers to identify focal seizure activity.

In summary, elevated hippocampal and cerebral cortex concentrations of cefepime are associated with neurotoxicity in rats with acute kidney injury. This rat model is promising for application in discovering the mechanistic cause of cefepime-induced neurotoxicity.

## SUPPLEMENTAL MATERIAL

Supplemental Material aac.01005-25-s0001.pdf Fig. S1 and Table S1.

ASM does not own the copyrights to Supplemental Material that may be linked to, or accessed through, an article. The authors have granted ASM a non-exclusive, world-wide license to publish the Supplemental Material files. Please contact the corresponding author directly for reuse.
