# Supplementary material for: Elevated hippocampal and cortical cefepime concentrations correlate with seizures in an acute kidney injury rat model
Source: Antimicrob Agents Chemother. 2026 Feb 17;70(4):e01005-25. doi: 10.1128/aac.01005-25 (PMC13041311; doi:10.1128/aac.01005-25)
Supplement: Supplemental Material — Fig. S1 and Table S1. [file aac.01005-25-s0001.pdf]

Supplementary Figures

Figure S1: Concentration-time profiles for (a) plasma, (b) cortex and (c) hippocampus. Individual fits in pink, population fits in green.

a)

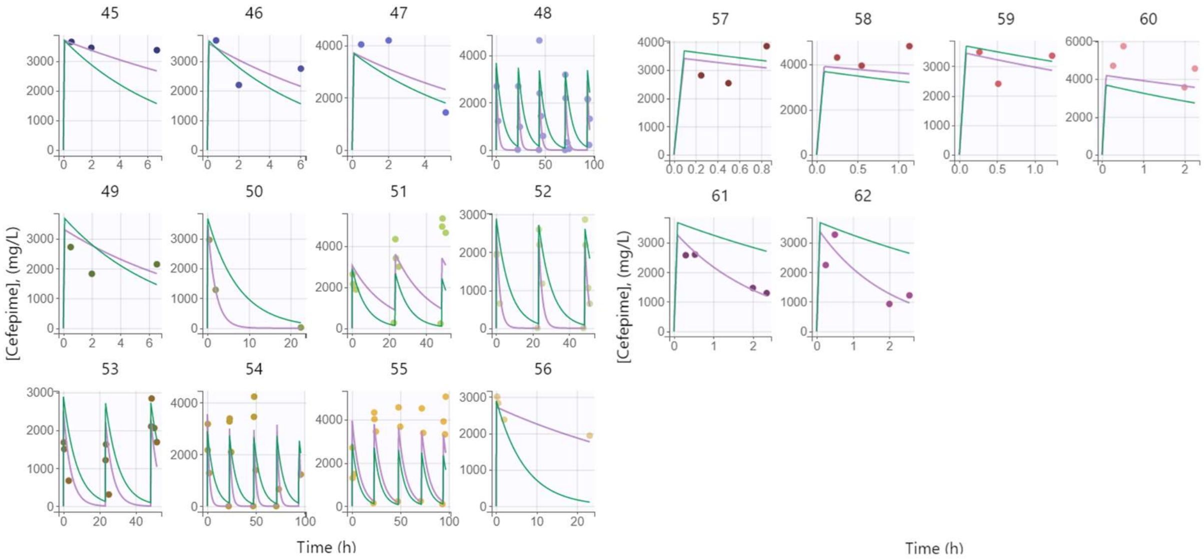

b)

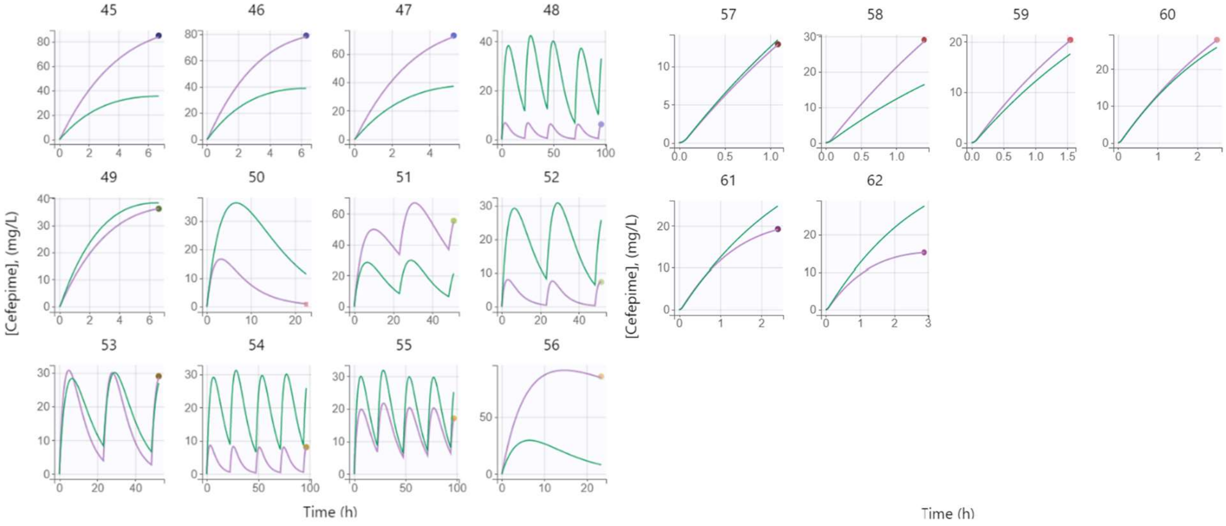

c)

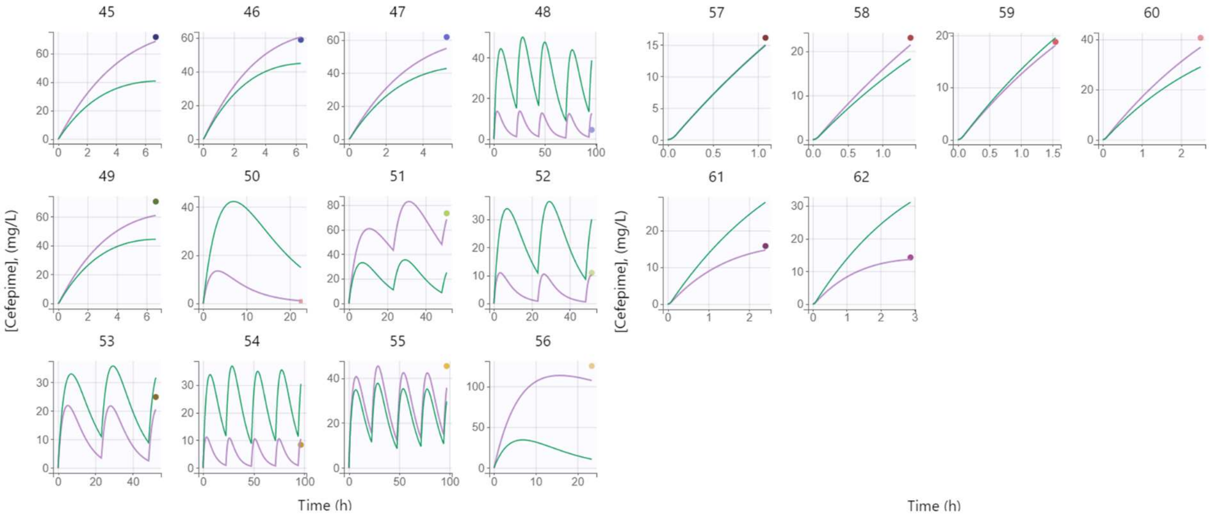
45
46
47
48
57
58
59
60
49
50
51
52
61
62
53
54
55
56
[Cefepime], (mg/L)
[Cefepime], (mg/L)
Time (h)
Time (h)

Supplementary Tables

Table S1. Calculated cefepime half-life in rats[10,14] & humans[1] with normal or impaired renal function, Mean (±SD)

	t½ (h)	
Renal function	Normal	Impaired
Rat	0.2 (0.2)	2.2 (1.1-5.8)
Human	2.0 (0.3)	13.5 (2.6), 19 (2)
